# Knowledge, Attitudes, and Practices of Antibiotic Use among Small-, Medium-, and Large-Scale Fish Farmers of the Stratum II of the Volta Lake of Ghana

**DOI:** 10.3390/antibiotics13070582

**Published:** 2024-06-23

**Authors:** Samuel O. Dandi, Emmanuel D. Abarike, Seth M. Abobi, Dzigbodi A. Doke, Jan L. Lyche, Samuel Addo, Regina E. Edziyie, Amii I. Obiakara-Amaechi, Evensen Øystein, Stephen Mutoloki, Kofitsyo S. Cudjoe

**Affiliations:** 1 Department of Aquaculture and Fisheries Sciences, Faculty of Biosciences, University for Development Studies, Tamale P.O. Box TL 1350, Ghana; sdandi@uds.edu.gh (S.O.D.); mabobi@uds.edu.gh (S.M.A.); 2 Department of Environment and Sustainability, Faculty of Natural Resources and Environment, University for Development Studies, Tamale P.O. Box TL 1350, Ghana; ddoke@uds.edu.gh; 3 Faculty of Veterinary Medicine, Norwegian University of Life Sciences, 1432 Ås, Norway; jan.l.lyche@nmbu.no (J.L.L.); oystein.evensen@nmbu.no (E.Ø.); stephen.mutoloki@nmbu.no (S.M.); 4 Department of Marine and Fisheries Sciences, School of Biological Sciences, University of Ghana, Accra P.O. Box LG 25, Ghana; samaddo@ug.edu.gh; 5 Department of Fisheries and Watershed Management, Faculty of Natural Resources, Kwame Nkrumah University of Science and Technology, Kumasi P.O. Box Up 1279, Ghana; reedziyie.frnr@knust.edu.gh; 6 Department of Marine Sciences, Faculty of Science, University of Lagos, Lagos 101017, Nigeria; ausese@unilag.edu.ng; 7 Norwegian Veterinary Institute, 1431 Ås, Norway; kofitsyo.cudjoe@vetinst.no

**Keywords:** antibiotics, knowledge, attitude, practice (KAP), cage aquaculture, volta lake, Ghana

## Abstract

Background: Antibiotic residue in food products and the resulting antibiotic-resistant bacteria represent a significant global public health threat. The misuse of antibiotics is a primary contributor to this issue. This study investigated the knowledge, attitudes, and practices (KAP) regarding antibiotic use among cage fish farmers on Ghana’s Volta Lake. Method: We conducted a cross-sectional survey with 91 cage fish farmers across three scales: small, medium, and large. A semi-structured questionnaire complemented by personal observations provided comprehensive data. We used several statistical methods for analysis: Pearson Chi-Square and Spearman correlation tests to examine relationships and trends among variables, logistic regression to analyze variable interactions, and Cronbach’s alpha to check internal consistency. Additionally, Kendall’s coefficient was used to rank challenges, utilizing STATA and SPSS for these calculations. Results: The survey revealed that 58.55% of cage fish farmers earn an average of 10,000 USD annually, with 35.16% having over 16 years of experience. From the survey, all sampled populations admitted to antibiotic applications in their farming operation. Knowledge of antibiotic types was mainly influenced by peers (46.15%), with tetracycline being the most recognized and used. There was a significant reliance on the empirical use of antibiotics, with 52.75% of farmers using them based on personal experience and 40.66% without a prescription. When initial treatments failed, 41.76% of the farmers would change or combine drugs. Older farmers (over 51 years) and those with tertiary education demonstrated significantly better KAP scores regarding antibiotic use. Strong correlations were also found among knowledge, attitudes, and practices in antibiotic usage. Conclusions: The findings indicate a need for improved education on antibiotic use among fish farmers to reduce misuse and enhance awareness of the potential consequences. This study provides foundational data for designing interventions to address these issues in the context of cage fish farming on Volta Lake.

## 1. Introduction 

Ghana’s tilapia and catfish aquaculture industry are crucial for economic growth, contributing about 5% to GDP. Cage aquaculture systems in Ghana consist of small-, medium-, and large-scale farms, accounting for approximately 90% of farmed fish, with the remaining 10% coming from other culture systems like ponds and tanks. The industry has experienced rapid growth in cage farming on Lake Volta, with an annual increase of 73%, making it the fastest-growing aquaculture industry in Ghana [1]. However, in late 2018, a disease outbreak caused by the infectious spleen and kidney necrosis virus (ISKNV) and bacterial infections resulted in substantial mortality of fish in cage farms in Lake Volta, followed by a decline in aquaculture production from 65,000 metric tons to 52,000 metric tons in 2019 [2]. The following burden of disease outbreaks, disease diagnosis, and the overall fish health management practices were mostly handled independently by the farmers and lacked a collaborative approach established by fish health authorities in Ghana on the Volta Lake [3]. 

It has been documented in many Asian aquaculture-intensive countries that aquaculture farmers use antibiotics and antibiotic-formulated feeds to treat and prevent all kinds of infections in fish farming as well as promote the fast growth of fish, boost fish immunity, and increase the survival of fish to maximize profit [4,5,6,7,8,9]. A review revealed the wide use of tetracyclines, oxolinic acid, sulfonamides, and erythromycin antibiotics in aquaculture production [5,8,10,11,12]. However, the use of antibiotics in animal feeds varies across geographic regions depending on the local food regulatory authorities, antibiotic usage profiles, monitoring systems, and even regulatory frameworks governing the use of antibiotics [13,14,15]. There has been an indication of the use of antibiotics in earthen and concrete fish ponds in Ghana [16,17,18]. A recent article revealed the presence of antibiotic residues in water and soil sediments sampled from cage farms of Volta Lake, Ghana [19,20]. Antibiotics, in general, are mostly limited to water solubility and resilience to deterioration and biodegradation [5]. Unregulated antibiotic use in fish culture has the tendency to increase resistant traits to both animal and human pathogens, affecting food quality and its safety by leaving residues in food products and, as well as its effect on organs like the liver, has become a major threat to public health and general animal welfare [5,20,21,22]. The One Health concept stresses reducing antibiotic resistance concerns at the human–animal–environment interface, with the goal of improving control mechanisms and addressing the complicated issue regarding antibiotic overuse in aquaculture [23,24,25,26,27]. Furthermore, occupational health hazards of aquaculture workers and human health risks connected with recreational waters posed by antibiotic resistance have been emerging issues but have been scarcely evaluated [28,29]. Some pathogenic bacteria, such as Aeromonas, play an important role in the human–animal–environment interface, and their ability to transmit tetracycline resistance under oxytetracycline stressors has been investigated [30,31]. Given the significance of cage fish farming to the local and national economy and the increasing call for ecologically friendly fish farming methods, antibiotic use, mode of application, and withdrawal period, as well as the role of stakeholders in the use of pharmaceuticals in fish farming on the Volta Lake needs to be evaluated and documented. This study, therefore, seeks to address the gaps in knowledge by examining the knowledge, attitude, and practices of cage farmers on antibiotic use and the role of stakeholders in regulating antibiotic use in cage aquaculture on the Volta Lake. 

## 2. Results

### 2.1. Demographic Characteristics of Respondents 

All the sampled farmers, 91 (100%), were males. Many of the farmers interviewed were in the age bracket of 50 years old and above (26.37%). The farmers had varied levels of education, with those without any form of education representing 28.57% being the majority. Out of the sampled population, 48.35% of the respondents indicated that they were owners of the farms (Table 1). 

### 2.2. Level of Experience in Fish Farming

Table 2 below shows that most farmers have been in the industry for at least 16 years and above, accounting for 35.16% of the sampled populations. According to the survey results, most current fish farmers, accounting for 41.76%, joined the industry to gather personal experience. About 58.25% of respondents indicated they earn an average income of 10,000 USD per year (Table 2).

### 2.3. Ranks of Challenges Facing Fish Farmers on the Volta Lake

The result of Kendall’s rank of coefficient of concordant analysis shows that the cost of feed, cost of fingerlings, mortality, and disease outbreaks are the most pressing and statistically significant (*p* = 0.001) problems faced by the farmers, as presented in Table 3 below.

### 2.4. Knowledge Level of Fish Farmers on the Use of Antibiotics 

#### 2.4.1. Most Common Groups of Antibiotic Drugs Used in Fish Farming on the Lake Volta 

The survey shows that fish farmers are familiar with the use of tetracycline, fluoroquinolones, amphenicol, and macrolides groups of antibiotics. Among these antibiotic groups, tetracycline group, representing 43.96% of all antibiotics, seems to be the most predominant among farmers compared to the amphenicol group of antibiotics, which accounted for 8.79%. The macrolide and fluoroquinolone groups accounted for 25.27% and 17.58%, respectively, while unspecified brands tagged “Others” accounted for 4.40%. 

#### 2.4.2. Sources of Information on the Use of Antibiotics in General in Fish Farming

**Figure 1:**
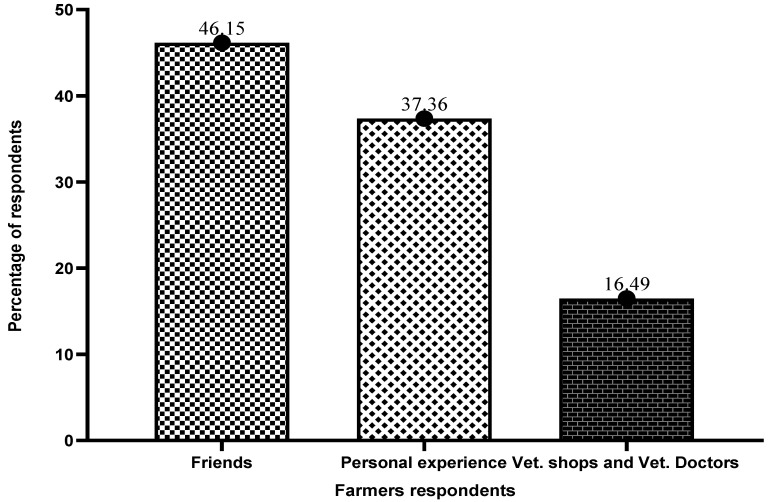
Sources of information on antibiotics in fish farming.

Figure 1 below presents information on the sources of information for farmers regarding specific antibiotic use. A total of 46.15% of respondents indicated that they learned about the application of any antibiotic groups in cage fish culture through their friends. Some respondents indicated they attempted to learn about antibiotics in fish farming through their encounters (37.36%), with veterinary shops and veterinarians (16.49%), representing the least source.

#### 2.4.3. Symptoms/Common Diseases That Affect Cage Fish Farms on the Lake Volta

Of all the symptoms/common diseases affecting cage fish farms on Lake Volta, responders noted ISKNV, Streptococcus, Columnaries infections, Whitish mouth, dark skin, rotten tail, swollen belly, bulgy eyes, and bent body were the most prevalent. Further to this, swollen belly, bulgy eyes, and bent body accounted for 43.96%, while ISKNV, Streptococcus, and Columnaries represented 13.19% of the most prevalent diseases affecting most farms in the Volta Lake. Among the multiple symptoms mentioned, most of the respondents (25.27%) identified whitish mouth, dark skin, rotten tail, and other infections (17.58%) as a common problem. 

#### 2.4.4. Perceived Reasons for Farmers’ General Use of Antibiotics in Fish Farming

Perceptions and views regarding antibiotic use in the Volta Lake region varied greatly among the respondents and sampled populations. The majority of the respondents, representing 41.76% of the sampled population revealed that the primary reasons behind using antimicrobials in fish culture are to increase survival, boost immunity, reduce mortality, enhance growth and digestion, and treat wounds. A considerable number of the respondents noted that antibiotics were used to slow down the severity of all infections; some thought of vaccination (19.78%), 15.38% perceived it to be a fast, quick, immediate, and cheap treatment method, while others indicated that human drugs work effectively in fish (13.19%) as well as being the only option available to boost fish immunity (9.89%). 

#### 2.4.5. Farmers’ Perceived Risk and Effect of Improper Use of Antibiotics in Fish Culture

The survey indicated that fish respond poorly to treatment and sometimes treatment fails when the correct dosage is not applied (34.07%). Death of fish, reduced feed intake, and water quality issues accounted for 31.89% of the perceived risk, while 18.68% perceived overdose of drugs, wastage of drugs, and waste of money to be a risk factor. Besides the 4.39% that indicated that they did not know, 10.99% indicates that improper use of antibiotics might result in resistance, dark skin, and change in gill color. 

### 2.5. Attitude of Cage Fish Farmers with the Use of Antibiotics in Fish Farming

#### 2.5.1. Sources of Information on Specific Antibiotics and Their Usage in Fish Farming

**Figure 2:**
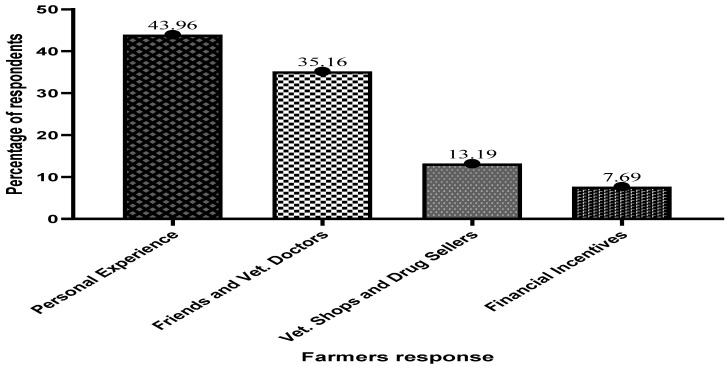
Information on specific antibiotics and their usage by fish farmers on Volta Lake.

Figure 2 below shows how fish farmers obtain and use specific antibiotics. The survey revealed that 43.96% of the respondents learned about antibiotics and their use through personal encounters and experiences gathered from other farms.

#### 2.5.2. Factors That Influence the Desire of CAGE Fish Farmers to Use Antibiotics

**Figure 3:**
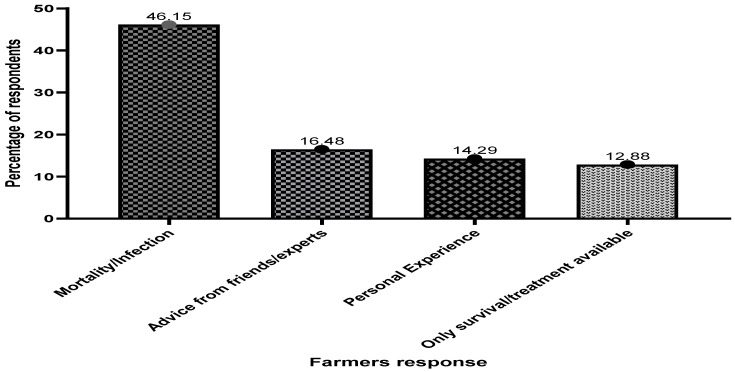
Factors that influence the desire of fish farmers to use antibiotics in fish culture.

Figure 3 depicts elements influencing farmers’ desire to use antibiotics in fish farming on Lake Volta. The survey shows that most cage fish farmers, accounting for 46.15% of the sampled population, are often persuaded or forced to use antibiotics due to high mortalities and diseases from unexplained causes.

#### 2.5.3. Suggested Ways to Reduce the Rate of Antibiotic Use in Fish Culture

**Figure 4:**
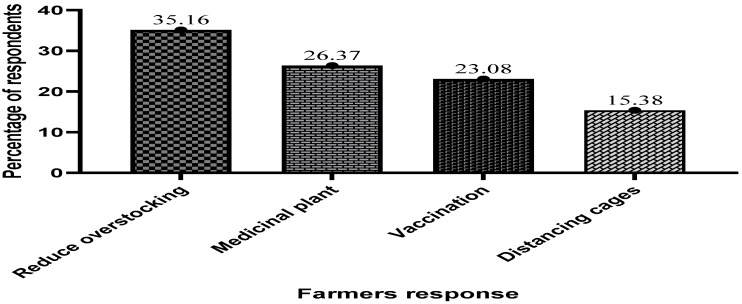
Farmers suggested ways of reducing the use of antibiotics in cage aquaculture.

Figure 4 below indicates some results on possible ways to reduce antibiotic use in fish culture, with the majority of respondents indicating that reducing stocking density, avoiding overfeeding, water quality monitoring, quality feed, and reducing stressful activities are some of the ways to help reduce the rate of antibiotic use in fish culture, accounting for 35.16% of all respondents. The need to explore the use of medicinal plants and cage separation was also emphasized.

#### 2.5.4. Perceived Role of Antibiotics in Fish Health Management

**Figure 5:**
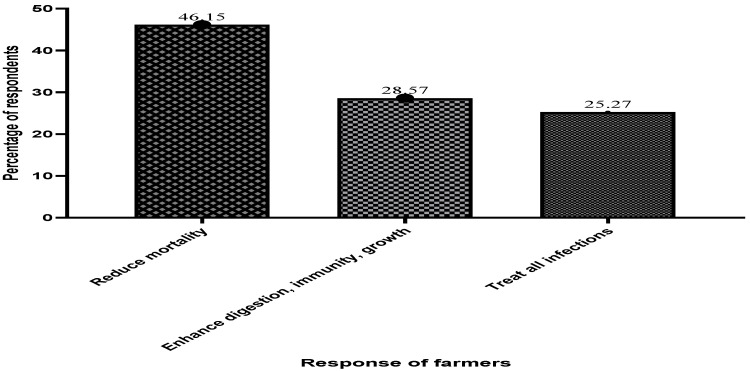
Cage fish farmers’ view on the role of antibiotics in fish health management.

Farmers, accounting for 46.15% of all respondents, stated that the most important role or function of antibiotic application in fish culture is to reduce the severity of any infection, increase fish survival, and reduce mortality from unknown causes. Reliability and effectiveness in treating all illnesses, stimulating appetite, and boosting immunity were attributed to antibiotics (see Figure 5 below).

### 2.6. Practice on the Use of Antibiotics in Fish Farming

#### 2.6.1. Farmers Suggested Ways to Prevent and Manage Fish Disease

**Figure 6:**
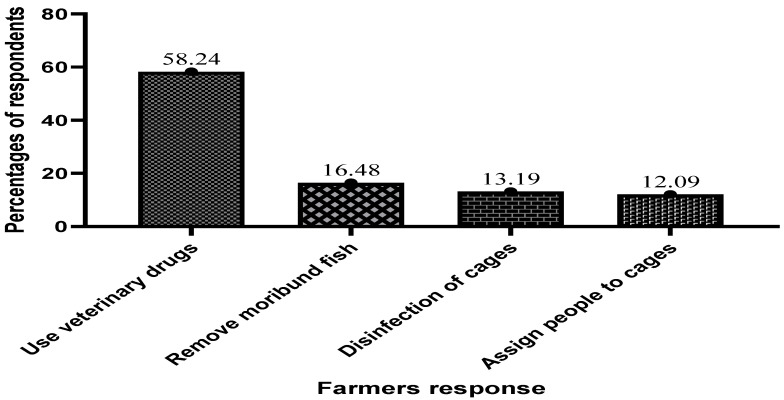
Precautions taken by cage farmers to prevent and manage diseases.

The survey shows that 58.24% of respondents agreed that they only manage the disease on their farms by using veterinary drugs. About 12.09% indicated they assign people to cages and use heat shock treatment (Figure 6).

#### 2.6.2. Ways Cage Farmers Handle Sudden Change in Fish Behavior 

**Figure 7:**
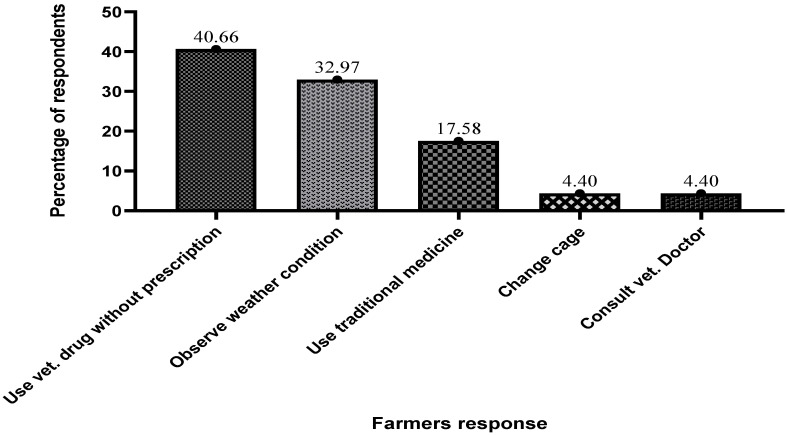
Approaches used by farmers to address sudden change in fish behavior.

According to the survey, most fish farmers sought to use veterinary drugs without prescriptions, representing 40.66% of the respondents (Figure 7). The results also revealed that observing weather conditions, using traditional medicines, and reducing feeding were the other methods employed by farmers (4.40%) to handle sudden changes in fish behavior.

#### 2.6.3. Chemical Drugs and Substances Used by Cage Fish Farmers to Treat Disease/Disinfect Farms

**Figure 8:**
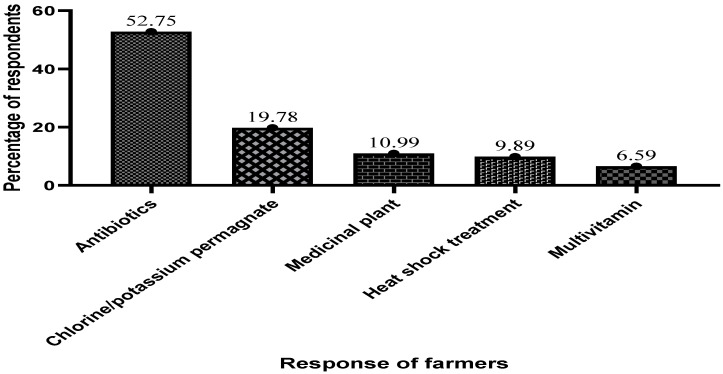
Chemicals farmers use in treating and disinfecting their cage farms.

Figure 8 below describes the substances and chemicals farmers use to treat and disinfect their cage fish farms. Out of the 91 respondents, the majority (52.75%) indicated that they use antibiotics in treating and disinfecting their farms.

#### 2.6.4. Reasons Cage Fish Farmers Use Antibiotics in Their Fish Farming Operations

**Figure 9:**
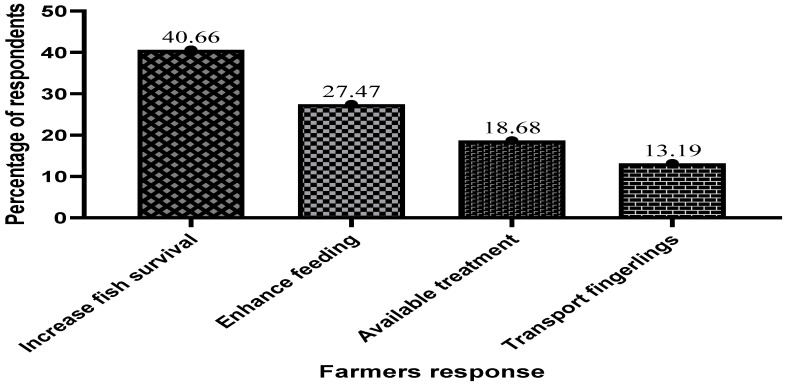
Reasons why fish farmers use antibiotics in fish farming.

Figure 9 below shows why cage fish farmers use antibiotics in their operations. About 40.66%, representing the majority of the respondents out of the sampled population, indicated that they use antimicrobials to increase fish survival, treat all infections in fish, reduce mortality from unknown sources, and treat wounds. Antimicrobials were also observed in fish culture to stimulate appetite or enhance digestion and were the only available treatment.

#### 2.6.5. How Cage Fish Farmers Apply Antibiotics in Fish Management

**Figure 10:**
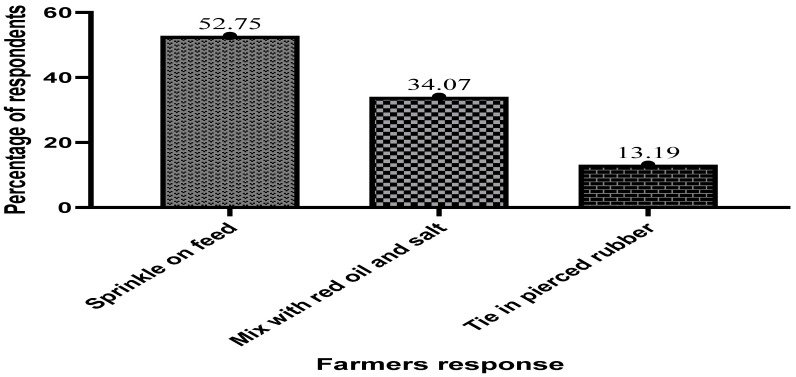
Mode of application of antibiotics in fish culture on Volta Lake.

The majority of the respondents (52.75%) indicated that they mostly mix or sprinkle antibiotics on the commercial feed. It was also observed that fish farmers normally mix the antibiotics with red oil and salt prior to mixing them with the feed, thereafter, the compounded antibiotics are tied in pierced rubber and onto the cage, as shown in Figure 10 below.

#### 2.6.6. Ways Farmers Judge the Effectiveness of Antibiotic Application in Fish Farming

**Figure 11:**
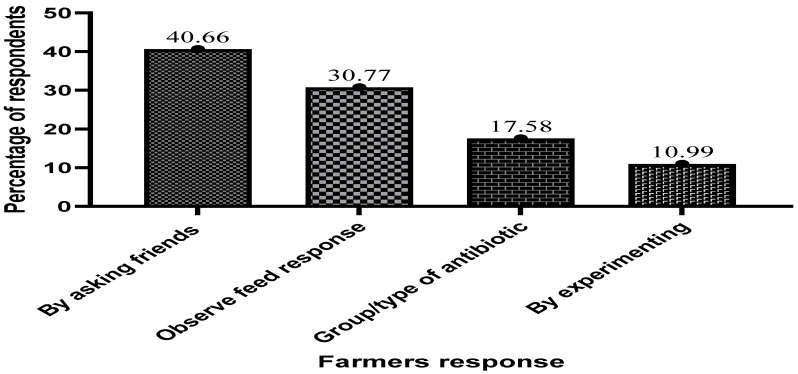
Ways farmers judge the effectiveness and efficacy of antibiotics in fish farming.

The results of how fish farmers judge the effectiveness and efficacy of antibiotics in fish farming are shown in Figure 11 below. The majority of the respondents, accounting for 40.66% of the surveyed population, indicated that they only judge the effectiveness of a drug by depending on friends who have used it before and by consulting experts in aquaculture.

#### 2.6.7. Reaction of Farmers after Antibiotic Application Proves Ineffective in Fish Farming

Evaluation of the data obtained from the survey showed that 41.76% of the sampled population either modified or combined most antibiotics if they applied them and did not receive the desired effects on their field. A number of the respondents, representing 31.87%, 14.76%, and 8.79% of the sampled population, indicated the addition of local salt and palm nut oil, seeking others for advice and changing of feeds, respectively, as some of the observed reactions of farmers towards the ineffectiveness of antibiotics. However, 3.40% stated that they did not know.

#### 2.6.8. Farmers Suggestions and Recommendation on the Use of Antibiotics in Farming

Table 4 presents some suggested recommendations from cage fish farmers on the use of antibiotics in fish culture. Most farmers (42.86%) agreed that sustainable antibiotic usage in fish culture might be achieved by collaboration, education, and training for farmers, medicine producers, distributors, and other stakeholders. The survey also revealed that counterfeit and expired pharmaceuticals are standard on the market, among others (Table 4).

### 2.7. Relationship between Knowledge, Attitude, and Practices of Fish Farmers

#### 2.7.1. Differences in Fish Farmers’ Knowledge, Attitudes, and Practices

The relevant relationships between the demographic data and the knowledge topic, as determined by the principal factor analysis, are shown in Table 5. From the results, age (*p* = 0.04), level of education (*p* = 0.016), role on a farm (*p* = 0.004), and number of cages (*p* = 0.002) are significant factors impacting the knowledge theme. The research found that the number of cages (*p* = 0.005), years of farming experience (*p* = 0.028), role on the farm (*p* = 0.017), and level of education (*p* = 0.007) were important factors influencing their attitudes. The research also found that a farmer’s age (*p* = 0.006), level of education (*p* = 0.036), years of farming (*p* = 0.001), and number of cages (*p* = 0.004) were the most essential characteristics influencing their practice (Table 5).

#### 2.7.2. Differences in Respondents’ Knowledge, Attitudes, and Practices

Table 6 shows the results of the logistic regression analysis on the demographic variables of farmers against their levels of knowledge, attitudes, and practices. Compared to other age groups, farmers with the age group of 51 years had 5.28 times the odds of having proper antibiotic knowledge (OR = 5.28, 95%; CIs = 1.13 8.70). In these multivariable logistic regression predictor models, it was discovered that farmers with tertiary education were 4.29 times more likely to have an acceptable understanding of antibiotic use (OR = 4.29, 95%; CIs: 1.11 7.47) than farmers with lower levels of education. Farmers with more than 41 cages are 1.066 times more likely to have an adequate understanding of antibiotic use in fish farming.

#### 2.7.3. Relationship between Knowledge, Attitudes, and Practices of Antibiotic Use

Significant variations were observed for the examined variables in the present study (Table 7). Spearman’s rank-order correlation revealed a positive association between respondents’ knowledge, attitude, and practice scores (*p* < 0.005). 

## 3. Discussion 

Global aquaculture has considerably increased the availability of fish for human consumption, reduced pressure on natural stocks, and employed many trained and unskilled individuals across the value chain [13]. However, it has also had a detrimental influence on the environment and public health, including disease outbreaks, environmental pollution, and residues of antibiotics in products [7,9,32,33,34]. Certain antibiotics are permitted and used in some countries either for the purpose of treating or preventing diseases in aquaculture [6,7,12,35,36]. Recent research revealed Vietnam as the top user of antibiotics in aquaculture between 2008 and 2018 [37], while Chile was deemed the country with the most significant antibiotic use per ton of fish harvested [38]. It is necessary to better understand the status of antibiotic use in all aquaculture systems, especially in cage aquaculture since the use of antibiotics in aquaculture varies between regions, countries, species, production phases (hatcheries, nurseries, and grow-out), and farming systems [6,7,27,39,40]. Little or no attention has been given to the assessment and effects of antibiotic use on freshwater cage aquaculture fish species for domestic trade. Cage aquaculture farms contribute to local and international food security, sustainable rural and urban development, job creation, and water efficiency. Antibiotic residues have been found in water and sediment samples from cage farms [20]. Similarly, antibiotic-resistant *A. hydrophila* and *Streptococcus* spp. strains have been reported in aquaculture systems in Volta Lake [24,41]. These secondary data support our findings in the current study, which show that antibiotics are widely used in cage aquaculture in Ghana. 

Misuse and lack of antibiotic awareness endanger antibiotic-resistant microbes, environmental and farmed fish residues, and human food security [7,9,12,38]. According to the survey, most fish farmers have been in the profession for 16 years or more and have an average income of 10,000 USD earned per year. Feed expenses, fingerling costs, mortality, and disease outbreaks are all issues that must be addressed as the significant challenges farmers face. On the other hand, most cage farmers believe that antibiotics can be used to treat any infections caused by any microorganism and that they may also be used to fatten fish quickly. A possible reason could be ascribed to the cheap and initial effectiveness of antibiotic application in disease control, and the general animal welfare and the tenacity of farmers to raise fish and to make a profit by all means within the shortest period. A similar study has been reported on freshwater fish and giant prawn (*Macrobrachium rosenbergii*) farmers in southern Vietnam, where these farmers reportedly use antibiotics for treating diseases and for prophylaxis purposes without any prior diagnostic tests [42,43]. The study revealed that a number of the farmers had been exposed to the tetracycline group of antibiotics, with the majority of them admitting to having learned about antibiotic usage from friends. These antibiotics increase survival, boost immunity, lower mortality, promote growth, improve digestion, and cure wounds. Common diseases caused by ISKNV and streptococcus predominantly affect most cage farms. The survey recorded the ineffectiveness of treatment and even death of fish due to inappropriate application of antibiotics by farmers. Despite this, farmers use or overuse antibiotics in cage aquaculture. The main reason could be a lack of information, training or access to alternative treatments or management practices as a replacement for antibiotics for treating infectious diseases in fish, in the form of vaccination, probiotics, or herbal/plant extracts. Also, other treatments, such as vaccination and probiotics, which have proven promising candidates to replace antibiotics, are also not specific to fish species and are costly. It was also revealed that there is no diagnostic center around the Lake that could respond to farmers in times of difficulty. Moreover, in the local environment, farmers tend to believe in the experience of friends in a related field rather than consulting experts on a particular issue regardless of its consequences. In a related study, it was noted that Vietnamese farmers reported treatment failure and poor response due to inappropriate antibiotic dosage, demonstrating a lack of awareness about the hazards of antibiotic usage in marine fish and lobster cage farming [39].

The survey revealed that fish farmers obtain either human or animal antibiotics through personal encounters without appropriate monitoring or regulation. Reducing antibiotic use in fish farming can be accomplished by lowering stocking density, avoiding overfeeding, providing quality feed, and minimizing stressful activities. Most farmers have worked in the aquaculture business on the Volta Lake for more than 16 years and they might have come across these drugs through personal experience. Some also indicated that they had worked on people’s farms before establishing their own farms. All these could influence their exposure and experience to antibiotic use in fish culture [39]. Most respondents believe all antibiotics, be they for human or veterinary use, have an essential role in overall fish health management. Antibiotics have been tested and proven effective for therapeutic, metaphylaxis, and prophylaxis purposes both in humans and in animals. Other studies have shown that human-use antibiotics in aquaculture fields have increased and proven effective due to their efficacy, low cost, and availability [39,42,44]. With these findings considered, researchers, fish farmers, and stakeholders would need to study, collaborate, and share practical alternative approaches to disease outbreaks, treatment, and the development of a dependable and trusted replacement for these antibiotic compounds in order to reduce their use in aquaculture successfully. These issues appear to be more challenging in cage and open aquaculture systems since it is more complicated, as well as significantly more expensive to apply biosecurity and meet the requirement of Good Aquaculture Practice (GAD) standards [6,42,43]. The government must continue to support novel research and educational programs on antibiotic use and its effects. To address these concerns, there is need to work collaboratively to improve advisory services and give training on the responsible use of antibiotics, as well as alternative disease prevention and mitigation techniques.

Moreover, respondents utilize veterinary medications without a prescription when there is a sudden change in fish behavior and for any purposes concerning fish health management. According to the survey, most cage farmers base their drug effectiveness decisions on recommendations from friends who have used them before, with a lot revealing the level of infection and route of antibiotic administration as grounds for antibiotic inactivity. Caged farmers also agreed to combine or switch to different antibiotics when they did not obtain satisfactory results after their application. They also typically acquire drugs from street sellers for any purpose in fish farming. This was attributed to the fact that diagnosing a disease that usually delays and no diagnostic center even to carry out a simple diagnosis, as well as no specific antibiotics for fish, are significant reasons why farmers depend on other farmers for the type, timing, and to judge the effectiveness of the antibiotic application. Easy and cheap access to medications from roadside sellers without interrogation also influenced farmers’ desire to acquire drugs from these areas rather than buying from veterinary shops which are usually occupied by veterinary protocols. This study agrees with other surveys conducted by [45,46]. It has been reported that farmers in the Mekong Delta of southern Vietnam who use higher doses of antibiotics do so based on personal experience rather than instructions [42,43]. Antibiotic usage in Ghana is primarily due to availability, convenience, and cost and is mostly for human and animal use. Therefore, governments and stakeholders of concern must impose more robust monitoring and restriction of antibiotic sales for aquaculture and for food producing animal users in general. Different nations have different distribution and registration systems and upper-middle- and high-income countries require veterinarian antibiotic prescriptions on antibiotic application [6,47]. 

Previous research has identified a knowledge gap in antibiotic misuse in resource-constrained aquaculture businesses and areas, with demographic factors significantly influencing knowledge, attitudes, and practice [6,39]. Previous studies discovered that age, education, farm type, and farm size were significant determinants of farmers’ KAP for antibiotic use in aquaculture farms [6,39]. According to the present study, age, level of education, and number of cages significantly (*p* < 0.05) influence proper knowledge of antibiotics use in cage aquaculture, while age, role on the farm, years in farming, and number of cages significantly (*p* < 0.05) influenced “desirable” attitudes in antibiotic use. Age, level of education, and number of cages significantly (*p* < 0.05) the odds of having “better” practice towards antibiotics use. The findings of several studies [48,49,50] are consistent with the current survey results. Regardless of their level of knowledge, attitudes, and practices, respondents engaged in inappropriate use of antibiotics in fish culture due to less supervision and surveillance [15]. The study finds a substantial relationship between farmers’ age and knowledge, attitudes, and practices on antibiotic use, with those farmers aged 51 years and above having better practices than younger farmers. This could be a result of the eagerness of the young generation to make a profit at all costs, thereby not following good husbandry practices, a conclusion comparable to a Bangladeshi study [44]. To combat the rate of antibiotic use in fish culture, human behavior and educational level are crucial [51]. In addition, to enhance the proper use of antibiotics, farmers must have a high level of education and adopt certain behaviors [52]. A farmer’s educational status is substantially linked (*p* < 0.05) with knowledge and practices regarding the general use of antibiotics [53]. The study also found that farmers with tertiary education showed better KAP responses toward antibiotic use, similar to a Turkish study emphasizing higher education about antibiotic usage [54]. Highly educated farmers have access to veterinary services, farm management, biosecurity measures, and a better grasp of antibiotic use and withdrawal times [55]. According to the study, farmers with low knowledge ratings were more likely to apply antibiotics inappropriately, which is similar to the findings of a study in Cameroon and Bangladesh, where farmers with lower knowledge were more likely to be untrained in poultry farming and therefore will not adhere to antibiotics protocols [56,57] and with drug and feed sellers reporting that farmers who received training had appropriate practices regarding AMU and AMR, respectively. The present study advocates that farmers be encouraged to participate in antibiotic training programs to create a baseline of knowledge. Educational campaigns, seminars, and mass media communications should be organized to train fish farmers with the assistance of physicians and veterinarians [51,56]. High costs of veterinary services, animal healthcare, feeding, and animal loss may promote negative attitudes and practices toward proper animal husbandry [58,59].

To reduce antibiotic use in cage aquaculture, farmers and stakeholders must have access to affordable disease diagnostic services closer to Volta Lake and be better informed about antibiotic resistance and other risks. Improving farm biosecurity can help avoid the possibility of disease outbreaks while also managing environmental cleanliness and water quality, particularly on Volta Lake [20]. Pre- and probiotics are widely used in aquaculture systems worldwide, particularly in intensive and superintensive forms, to prevent and manage disease outbreaks while maintaining water quality [60,61]. Vaccination has also proven beneficial in lowering antibiotic use in aquaculture [38,62,63]. Ghana’s tilapia and cage culture systems are still in the early stages of development compared to European countries due to a lack of appropriate systems to check vaccines, delivery systems, and costs. Corporate–public collaborations between the government, academia, and the corporate sector can help to provide low-cost and customized immunizations that can all help to reduce antibiotics in cage aquaculture.

## 4. Materials and Methods

### 4.1. Ethical Consideration and Approval

All ethical clearance and consent were sought and obtained concerning the present study from the University for Development Studies and the Ministry of Fisheries and Aquaculture Development, Ghana (REF. NUMBER FC 0.5/9) 

### 4.2. Study Area

**Figure 12:**
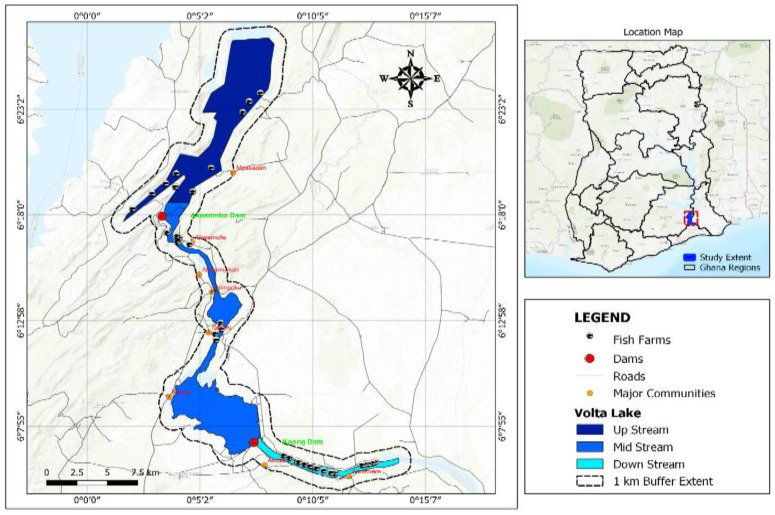
Map of the stratum II of the Volta Lake, Ghana showing the sampling site.

Sampled cage farms in stratum II of the Volta Lake within the Akosombo Dam at 6°17′57.7″ N 0°03′19.6″ E is designated as upstream, through the area between the Akosombo Dam and Kpong Dam at 6°07′12.4″ N 0°07′31.4″ E is designated as midstream, and after the Kpong Dam at 6°06′02.3″ N 0°09′26.2″ E through to the Akuse area is designated as downstream. These demarcations are the original sections of the stratum II of the Volta Lake, which were the sampled areas for the study (Figure 12) [64].

### 4.3. Development and Pretesting of Research Instrument

A multidisciplinary group of aquaculture professionals, environmental scientists, drug producers, pharmacists, food scientists, water quality experts, fish farmers, and consumers collaborated to design a questionnaire on antibiotic use in cage aquaculture systems. The questionnaire focused on farmers’ demographic characteristics, level of experience in fish farming, knowledge, attitude, practice, personal experience, and some recommendations on the use of antibiotics in fish farming, as well as the role of veterinarians and government fisheries officials in the use of antibiotics in fish culture on the Volta Lake. The questionnaire was complemented by physical observations of antibiotic application and practice in cage farms to obtain more in-depth information about the study. The questionnaire was pilot-tested between October and November 2023 with a sample of the target group. Appropriate measures were taken to preserve and maintain confidentiality, anonymity, and voluntarism throughout the study.

### 4.4. Sampling Techniques and Design

Purposive sampling was used in choosing fish farmers along the strata for the study, from December 2023 to February 2024. In each designated site, ten (10) or eleven (11) small-scale (i.e., farms with cage dimensions 5 m × 5 m × 5 m), medium-scale (i.e., farms with cage dimensions 6 m × 6 m × 6 m), and large scale (i.e., farms with cage dimensions 12 m × 12 m × 12 m) individual cage fish farms with a general stocking density between 6000–200,000 fingerlings/grow out per cage. In all, a total of 91 (30 small-scale, 30 medium-scale, and 31 large-scale farms) individual cage fish farms were sampled.

### 4.5. Data Analysis

All data obtained from the field, including demographics, level of experience in fish farming, knowledge, attitude, and practice about antibiotic use, were coded into a computerized database and analyzed using STATA software version 17. In all, 91 cage fish farmers of valid data cases were incorporated and analyzed, and the results were presented as frequencies and percentages in tables and charts. Pearson’s chi-square test was used to determine whether the observed variables are related to each other, Spearman’s correlation was used to assess the strength and direction of the tested variables, logistic regression was used to analyze the relationship between the tested variables, and validity and liability tests were done by using Cronbach’s alpha to check for internal consistency which was all performed using STATA. In contrast, Kendall’s coefficient of concordant was carried out using SPSS version 24 to rank the challenges faced by farmers.

## 5. Conclusions

The study revealed inadequate knowledge, attitude, and inappropriate practices as significant threats to the misuse of antibiotics in fish farming. This research will support further policy formulations and strategies to educate, train, and create awareness regarding antibiotic use in fish farming. It will also be prudent to monitor antibiotic sellers, train all stakeholders on drug use in fish farming, and refine and help implement local and national antibiotic action plans and efforts to control, regulate, and monitor antibiotic use in fish farming. A vital component of this antibiotic action plan will require the promotion of education and training towards attaining one health approach and effective collaboration in tackling the current misconception about the use of antibiotics. Setting up simple diagnostic centers around more intense fish farming areas, enforcing monitoring, collaborating, and enacting strong antibiotic prescription legislation in Ghana to minimize illegal, unreported, and unregulated widespread use of antibiotics are strongly recommended. 

## 6. Recommendation

To attain a sustainable cage aquaculture industry, it is recommended that policies regarding antibiotics use in fish culture be developed, enforced, and practiced in an eco-friendly, collaboratively accepted, practically oriented, and inclusive way.

## Figures and Tables

**Table 1:** Demographic characteristics of respondents.

Characteristics	Category	Frequency	Percentage
Age	Less than 20 Years	1	1.10
	21–30 years	20	21.98
	31–40 Years	24	26.37
	41–50 years	22	24.18
	50 years and above	24	26.37
Level of Education	Tertiary	7	7.69
	Primary	13	14.29
	SHS	21	23.08
	JHS/JSS	24	26.37
	None	26	28.57
Role on the farm	Owner	44	48.35
	Manager	27	29.67
	Worker	20	21.98
	Others	0	0.00
	Total	91	100.00

Legend: JHS—Junior High School; JSS—Junior Secondary School; SHS—Senior High School.

**Table 2:** Experience level of fish farmers in cage aquaculture.

Characteristics	Category	Frequency	Percentage
Year in fish farming	Less than 1 year	0	0.00
	1–5 years	9	9.89
	6–10 years	22	24.18
	11–15 years	28	30.77
	16 years and above	32	35.16
Reasons for fish farming	Easy to manage	7	7.69
	Less starting capital	8	8.79
	Free water resources	10	10.99
	Trained personnel	13	14.29
	Supplementary income	15	16.48
	Gather personnel experience	38	41.79
Average income per year (Converted from Ghana cedis to US dollars)	Less than 1000 dollars	3	3.30
	2000 dollars	14	15.38
	5000 dollars	21	23.08
	10,000 dollars and above	53	58.25
Number of cages (5 m by 5 m, 6 m by 6 m and 12 m by 12 m)	Less than 10 cages	7	7.69
	11–20 cages	14	15.38
	21–30 cages	15	16.48
	31–40 cages	23	25.27
	Above 41 cages	32	35.16
Total		91	100.00

**Table 3:** Challenges faced by farmers.

Challenges	Mean Rank	Rank
Cost of feed, cost of fingerlings, mortality, disease outbreak	1.79	1st
Dam spillage, increase in water level, water pollution	2.13	2rd
Inadequate fingerlings, theft, wind	3.03	4nd
Lack of fish health personnel and market, swollen belly	2.77	3th
N	91	
Kendall’s Wa	0.771	
Chi-square	152.451	
df	3	
Asymp. Sig.	0.001	

**Table 4:** Farmers proposed recommendations on the use of antibiotics in fish farming.

Recommendation on the Use of Antibiotics	Frequency	Percentages
Research into the combination of red oil and local salt with antibiotics	8	8.79
Research into medicinal plants	9	9.89
Normally buy from vet shops	15	16.48
Screen the market for fake and expired ones	20	21.99
Collaboration, education, and training	39	42.86
Total	91	100.00

Legends: Vet—Veterinary.

**Table 5:** Test the statistical significance of variation in the respondents’ knowledge of antibiotic use based on their characteristics.

		Knowledge		Chi-Square	Attitude		Chi-Square	Practice		Chi-Square
Variable	Category	More Knowledge	Less Knowledge	*p* Value	Adequate	Inadequate	*p* Value	Advance	Unadvanced	*p* Value
	21–30 years	17 (18.68)	4 (4.39)		4 (4.39)	5 (5.49)		9 (9.89)	12 (13.18)	
Age	31–40 years	20 (21.97)	3 (3.29)	0.042	10 (10.98)	18 (19.78)		5 (5.49)	19 (20.87)	0.006
	41–50 years	19 (20.87)	3 (3.29)		14 (15.38)	18 (19.78)	0.89	13 (14.28)	9 (9.89)	
	51 years and above	24 (26.37)	4 (4.39)		8 (8.79)	14 (15.38)		9 (9.89)	15 (16.48)	
	None	22 (24.17)	4 (4.39)		2 (2.19)	24 (26.37)		20 (21.97)	6 (6.59)	
	Primary	12 (13.18)	4 (4.39)	0.016	3 (3.29)	10 (10.98)		12 (13.18)	1 (1.09)	
Level of education	JHS/JSS	19 (20.97)	1 (1.09)		9 (9.87)	15 (16.48)	0.007	18 (19.78)	6 (6.59)	0.036
	SSS	18 (19.78)	3 (3.29)		8 (8.79)	13 (14.28)		18 (19.78)	3 (3.29)	
	Tertiary	4 (4.38)	3 (3.29)		1 (1.09)	6 (6.59)		4 (4.39))	3 (3.29)	
	Manager	24 (26.37)	3 (3.29)		17 (18.68)	10 (10.98)		22 (24.17)	5 (5.49)	
Role of farmer	Owner	38 (41.75)	5 (5.49)	0.002	40 (43.95)	4 (4.39)	0.017	44 (48.35)	1 (1.09)	0.68
	Worker	14 (15.38)	6 (6.59)		15 (16.48)	5 (5.49)		19 (20.87)	1 (1.09)	
	1–5 years	1 (1.09)	8 (8.79)		3 (3.29)	6 (6.59)		7 (7.69)	2 (2.19)	
	6–10 years	11 (12.08)	17 (18.68)		4 (4.39)	24 (26.37)	0.028	24 (26.37)	4 (4.39)	0.001
Years in farming	11–15 years	8 (8.79)	24 (26.37)	0.19	7 (7.69)	25 (27.47)		30 (32.96)	2 (2.19)	
	16 years	4 (4.39)	18 (19.78)		8 (8.79)	14 (15.38)		11 (12.08)	11 (12.08)	
	11–20 cages	31 (34.06)	1 (1.09)		4 (4.39)	28 (30.76)		28 (30.76)	4 (4.39)	
Number of cages	21–30 cages	12 (13.18))	3 (2.29)	0.002	1 (1.09)	14 (15.38)		13 (14.28)	2 (2.19)	
	31–40 cages	6 (6.59)	1 (1.09)		2 (1.19)	5 (5.49)	0.005	6 (6.59)	1 (1.09)	0.004
	Above 41 cages	19 (20.87)	4 (4.39)		12 (13.18)	11 (12.08)		13 (14.28)	10 (10.98)	
	Less than 10 cages	12 (13.18)	2 (2.19)		3 (3.29)	11 (12.08)		12 (13.18)	2 (2.19)	

**Table 6:** Logistic regression analysis of the factors associated with respondents’ knowledge, attitudes, and practices of antibiotic use.

		Knowledge			Attitude			Practice		
Variable	Category	Odd Ratio (Exp B)	95% CL		Odd Ratio (Exp B)	95% CL		Odd Ratio (Exp B)	95% CL	
			Lower	Upper		Lower	Upper		Lower	Upper
Age	Less than 20	1.85642	0.96212	3.811554	0.11742	0.18562	0.92375	0.13968	0.18943	0.02614
	21–30	3.44786	0.9972	2.6684	0.02621	0.02314	0.39671	0.17746	0.2931	0.06521
	31–40	1.424719	0.96212	3.811554	0.00066	0.29329	0.291962	0.16173	0.3374	0.01395
	41–50	2.297162	0.14932	4.743642	0.106475	0.19346	0.406413	0.15877	0.3388	0.02130
	51 years	5.284593	1.13327	8.70245	4.104708	0.19172	3.887431	6.17358	1.3515	6.0439
Education	Primary	0.14375	0.70611	3.569419	0.077901	0.24214	0.39794	0.04483	0.2369	0.1473
	Jhs/jss	0.92742	0.8694	3.55371	0.29713	0..69487	0.92172	0.06341	0.1827	0.2694
	Sss	0.05114	2.11804	2.015754	0.154558	0.09884	0.407959	0.04829	0.2004	0.1038
	Tertiary	4.29033	1.11021	7.47202	3.047702	1.00218	5.437583	5.143383	1.0906	4.3774
	None	1.431655	0.70611	3.569419	0.175682	0.08641	0.437771	0.005876	0.1514	0.16322
Role on farm	Owner	0.359082	1.69931	2.417471	0.25953	0.51189	0.00717	0.22451	0.37602	0.07300
	Manager	0.92471	1.0942	1.00421	0.18952	0.22616	0.3651	0.31859	0.02461	0.00417
	Worker	1.53713	3.7771	0.702842	0.05397	0.32858	0.220654	0.15503	0.31991	0.98450
Years in farming	<1 yr	0.06917	1.7793	2.36214	0.3891	0.49706	0.85312	0.03142	0.08321	0.33143
	1–5	0.9392	2.2183	3.4491	0.0893	0.27933	0.6931	0.05531	0.08427	0.34407
	6–10	1.115312	1.63954	3.870164	0.03444	0.3033	0.372183	0.065125	0.13765	0.2679
	11–15	0.58398	3.40063	2.232674	0.187978	0.15734	0.533298	0.124113	0.1121	0.3384
	16 yr and above	0.08111	2.99085	2.828629	0.165837	0.1909	0.522569	0.129902	0.3812	0.6924
Number of cages	Less than 10	0.09487	1.35438	2.164636	0.00738	0.2844	0.269632	0.077867	0.32671	0.18923
	11–20	0.07241	0.9774	1.9558	0.0497	0.0851	0.17932	0.59421	0.4491	0.6342
	21–30	0.32829	2.6761	2.01951	0.007317	0.28052	0.295157	0.15389	0.1828	0.15513
	31–40	0.97924	1.99972	3.958204	0.12375	0.48897	0.241472	0.02793	0.08845	0.24182
	Above 41	1.06652	1.20092	3.33395	0.47384	0.75183	0.19586	0.01639	0.1382	0.97782

Legend: CL—Confidence Level.

**Table 7:** Spearman’s correlation between knowledge, attitude, and practice.

Variable	Correlation Coefficient	*p*-Value
Knowledge vs. Attitude	0.9394	0.0005
Attitude vs. Practice	0.8743	0.0045
Knowledge vs. Practice	0.9157	0.0014

## Data Availability

All data has been included in the manuscript.
